# Effects of Dietary Fat Sources during Late Gestation on Colostrum Quality and Mammary Gland Inflammation in Lipopolysaccharide-Challenged Sows

**DOI:** 10.3390/ani10020319

**Published:** 2020-02-18

**Authors:** Tiande Zou, Wenzhuo Wei, Shanchuan Cao, Hongfu Zhang, Jingbo Liu

**Affiliations:** 1 School of Life Science and Engineering, Southwest University of Science and Technology, Mianyang 621010, China; tiandezou@jxau.edu.cn (T.Z.); weiwenzhuo0616@163.com (W.W.); lecaoshanchuan@126.com (S.C.); 2 Jiangxi Province Key Laboratory of Animal Nutrition, Engineering Research Center of Feed Development, Jiangxi Agricultural University, Nanchang 330045, China; 3 State Key Laboratory of Animal Nutrition, Institute of Animal Sciences, Chinese Academy of Agricultural Sciences, Beijing 100000, China; zhanghongfu@caas.cn

**Keywords:** colostrum, fat, inflammatory response, lipopolysaccharide, mammary gland

## Abstract

**Simple Summary:**

In the present study, late gestating sows were challenged with lipopolysaccharide (LPS) endotoxin, which can impair the immune system of mammary gland cells and result in an inflammatory response. Additionally, the LPS-treated sows were fed 3% soybean oil (SO), 3% coconut oil (CO) or 3% fish oil (FO) diets and were used to study the effect of fat sources on the colostrum quality and mammary gland inflammation of sows exposed to immune challenge. The results show that FO inclusion exerted anti-inflammatory effects in mammary glands and counteracted the LPS-induced damaged colostrum synthesis and pro-inflammatory response when compared to CO diets. These findings suggest that fatty acid profiles of different oil types in late gestation differentially affect metabolic health in sows, but a longer period of FO supplementation to sows is needed to determine a positive effect on piglets.

**Abstract:**

This study aimed to investigate the effects of maternal lipopolysaccharide (LPS) challenge and dietary fat sources on colostrum quality and inflammatory response in sows. Sixty Landrace × Yorkshire sows were randomly assigned to three dietary treatments supplemented with 3% soybean oil (SO), 3% coconut oil (CO) or 3% fish oil (FO), respectively, from Day 90 of gestation until parturition. On Day 112 of gestation, half the sows from each dietary treatment were challenged with LPS (10 μg/kg BW) or saline. The results showed that maternal LPS challenge decreased colostrum yield and dry matter content. A similar pattern of changes was observed for body weight gain and colostrum intake in piglets from LPS-challenged sows. Maternal LPS challenge increased the levels of tumor necrosis factor α (*TNFα*), interleukin 1β (*IL1β*) and *IL6* in colostum, and the mRNA abundance of *IL6*, *IL1β* and *TNFα* and the phosphorylation level of *p65* in mammary glands. However, the responses of these variables to LPS treatment were lower in sows fed a FO diet. In conclusion, maternal immune challenge reduced the growth performance of piglets by decreasing colostrum yield and intake by piglets, and dietary supplementation with FO in sows attenuates the LPS-induced inflammatory response in mammary glands.

## 1. Introduction

The offspring growth and immunity from birth to weaning is affected by the maternal colostrum yield and quality, which contains numerous immunoglobulins and cytokines that are beneficial to newborn animals [1]. As maternal colostrum yield and quality are affected by diets, maternal nutritional intervention during gestation may provide an effective strategy to improve offspring growth and immunity. Fat plays an important role in sow nutrition, as its high energy value and low heat increment provides most of the energy as well as the fatty acids needed for the growth and maintenance of organ systems and the body’s energy storage in offspring [2]. More recently, supplementation of fat to the sow diets in late gestation and lactation was found to influence the fatty acid composition of colostrum and milk, modulate sow oxidative stress levels and inflammatory responses, and improve the survival of piglets [3,4,5]. Sow dietary fat sources, which differ in fatty acid composition, have different effects on sow colostrum quality and immunity. The present experiment selected three oil types, including soybean oil (SO), coconut oil (CO) and fish oil (FO). Fish oil is rich in omega-3 polyunsaturated fatty acids (n-3 PUFAs), and n-3 PUFAs have been shown to exert multiple beneficial effects, including lipid metabolism modification, oxidative stress and inflammation [5,6,7]. Coconut oil mainly contains medium-chain fatty acids (MCFAs) in proportions of more than 60%. Soybean oil is rich in 18-chain fatty acids such as C18:1 (n-9), C18:2 (n-6) and C18:3 (n-3). Unlike the proinflammatory role of CO and anti-inflammatory role of FO in the modulation of the innate immune toll-like receptor-4 signaling pathways, the soybean oil was neutral for toll-like receptor-4/NFκB signaling and could be used as a control of FO and CO [8]. In recent years, much attention has been focused on the consequences of modifying the fatty acid profiles of sow diets by dietary supplementation with different fat sources. To our knowledge, direct evidence that dietary n-3 PUFA inhibits inflammation in mammary gland of sows has not been provided yet.

Bacterial infection occurring during gestation can impair the immune system of mammary gland cells and result in an inflammatory response, which may play a negative role in the yield and composition of colostrum and milk [9]. Bacterial lipopolysaccharide (LPS) endotoxin has been widely used to develop inflammation in many animal species [10,11,12]. The LPS-induced activation of the inflammasome axis is important for the development of mammary gland injury. As n-3 PUFAs have potent anti-inflammatory properties [6], their inclusion in sow diets could protect against bacterial infection-induced oxidative stress and inflammation in mammary gland. However, not much is known regarding whether feeding FO to sows as a source of n-3 PUFA could attenuate LPS-induced sow stress levels and inflammatory responses.

Therefore, the present study aimed to test the two hypotheses: first, the pro-inflammatory process in mammary gland of late-gestating sows challenged with LPS leads to decreased reproductive performance and colostrum synthesis. Second, dietary supplementation with FO may exert anti-inflammatory effects in mammary gland of sows and thus counteract the LPS-induced pro-inflammatory response.

## 2. Materials and Methods

### 2.1. Ethical Statement

The experiment was performed according to the Chinese guidelines for animal welfare, and all experimental procedures of animal use were permitted by the Ethical committee of Southwest University of Science and Technology (permit No.1020130053) [13].

### 2.2. Animals, Diets and Experimental Design

A total of sixty multiparous sows (Landrace × Yorkshire; 3 to 5 of parity) were randomly selected and assigned into three dietary treatment groups (3% SO, 3% CO and 3% FO) based on backfat thickness and body weight (n = 20 per group) using a randomized complete block design. The dose of 3% was included as previously described, which showed beneficial effects of FO on the hypothalamic-pituitary-adrenal function in sows and offspring [14]. All sows in the present experiment were artificially inseminated by pooled semen from three Duroc boars. The ingredients and composition of the experimental diets and the analyzed fatty acids profiles of the fat sources are shown in Table 1 and Table 2, respectively. All diets were formulated to meet or exceed the nutrient requirements of gestating sows as recommended by the National Research Council (2012) [15]. All sows were housed in individual gestation stalls in a breeding facility and fed a daily ration of 2.5 kg of the experimental diets from Day 90 of gestation until parturition. The daily feed allowance of each sow was divided into 2 equal meals that were fed at 08:00 h and 14:00 h. The sows had free access to drinking water. On Day 110 of gestation, the sows were transferred to a farrowing room and housed in individual farrowing crates. On Day 112 of gestation at 08:00 h, 10 challenged sows per dietary treatment were injected intramuscularly with LPS (Sigma-Aldrich Co., St. Louis, MO, USA) from *Escherichia coli* strain O55:B5 (10 μg/kg BW) or sterile saline. Mammary gland tissues were collected under regional anesthesia at 24 h after LPS challenge and immediately frozen in liquid nitrogen until further analysis. In the current study, mammary secretions during the first 24 h after the birth of the first-born piglet, followed by the secretion of transient milk until Day 4 of lactation, were measured. Colostrum samples at 12 h postpartum were collected according to a previously described method [16] and the onset of transition milk was recorded. During parturition, the duration of farrowing was defined as the time between the birth of the first and last piglet. Piglets were weighed at birth and after the colostrum period. Colostrum intake by piglets from birth to 24 h after the onset of parturition was estimated based on a prediction equation, as previously described [17,18]. The onset of parturition was designed as the time of birth of the firstborn piglets detected by camera. The colostrum yield of each sow was calculated by summing the intake from each piglet within a litter.

### 2.3. Analysis of the Composition and Cytokines Concentrations in Colostrum

The composition of colostrum was measured using an automatic milk analyzer (Milk-Yway-CP2, Beijing, China). The concentrations of cytokines including interleuckin-6 (*IL6*), interleukin-1β (*IL1β*) and tumor necrosis factor-α (*TNFα*) were measured using the porcine-specific ELISA kits (R&D Systems, Minneapolis, MN, USA) according to the manufacturers’ instructions [19,20,21].

### 2.4. Quantitative Real-Time PCR Analysis

The total RNA was extracted from mammary gland tissues using TRIzol reagent (Sigma, Saint Louis, MO, USA). The purity and concentration of RNA was estimated by the Nanodrop ND-1000 (Nanodrop Technologies, Thermo Scientific, Wilmington, DE, USA). The integrity of RNA was examined by electrophoresis in agarose gel (1%). Following RNA extraction, cDNA was synthesized from 500 ng of total RNA with the iScript^TM^ cDNA Synthesis Kit (Bio-Rad, Hercules, CA, USA). Quantitative real-time PCR was performed on the CFX96 RT-PCR detection system (Bio-Rad, Laboratories, Hercules, CA, USA) with SYBR green detection, as in the procedure described previously [22]. TATA-binding protein (*TBP*), DNA topoisomerase Ⅱ beta (*TOP2B*), and β-actin (*ACTB*) were used as reference genes to normalize the gene expression data. The relative mRNA expression of the target genes was calculated using the method of 2^−ΔΔCt^, as previously described [23]. The primer sequences of the target genes and reference genes are shown in Table 3.

### 2.5. Immunoblotting Analysis

Immunoblotting analysis was conducted as previously described [24,25]. Briefly, protein extracts from mammary glands were separated by 10% SDS-PAGE gels and transferred to a polyvinylidene difluoride (PVDF) membrane (Millipore, Billerica, MA, USA). The membrane was washed with TBS with 0.1% Tween 20 (TBST) and incubated with 5% nonfat dried milk blocking solution at room temperature for 1 h, followed by incubation with primary antibodies (1:1000) overnight at 4 °C. The membranes were washed with TBST and incubated with a horseradish peroxidase (HRP)-conjugated secondary antibody for 1 h at room temperature. The densities of bands were quantified by the Image Lab statistical software (Bio-Rad, Laboratories, Hercules, CA, USA) and normalized to ACTB content [26].

### 2.6. Statistical Analysis

Statistical analysis was performed using the general linear model procedures of the SAS 9.1 software (SAS Institute, Cary, NC, USA). The statistical model included the fixed effects of maternal diet (SO, CO or FO), maternal LPS status (LPS or saline) as well as their interactions. The body weight and backfat of sows were the random effects in the model. The individual sow was considered as the experimental unit for each trait. All data were expressed as the mean values and standard error of the mean (SEM). A significant difference was considered as *p* < 0.05.

## 3. Results

### 3.1. Performance Characteristics

No significant differences between treatments were observed for body weight and backfat thickness in sows at Day 90 of gestation (Table 4). Similarly, there were no significant effects of LPS challenge and dietary oil type on the duration of farrowing, onset of transient milk, the number of piglets born alive, the mortality of piglets from Day 1 to 5 and the weights of piglets at birth (Table 4). Regardless of the maternal diets, the average weight gain of piglets from LPS-challenged sows was decreased compared to the saline-treated group (*p* < 0.01, Table 4).

### 3.2. Colostrum Production and Composition

As shown in Table 5, the colostrum yield and intake by piglets were not influenced by dietary oil type. Regardless of the maternal diets, the colostrum yields in LPS-challenged sows were lower than in saline-treated sows (*p* < 0.05). Dry matter content in colostrum was decreased by LPS challenge (*p* < 0.01, Table 6). A similar pattern of changes was observed for colostrum intake by piglets from LPS-challenged sows (*p* < 0.01). However, the responses of these variables to LPS treatment were lower in sows fed FO or SO diets. Moreover, there were no significant effects of LPS challenge and oil type on the contents of protein, fat and lactose in colostrum (Table 6).

### 3.3. Cytokine Productions in the Colostrum

The concentrations of pro-inflammatory cytokines *TNFα*, *IL1β* and *IL6* in colostrum were affected by the interactive effect of oil type and LPS (*p* < 0.01, Table 7). The LPS challenge increased the contents of *TNFα*, *IL1β* and *IL6* in colostrum for sows fed the CO diet. However, no effects of LPS challenge on these variables were found for sows fed the FO diet.

### 3.4. mRNA Abundances and Protein Expression of Inflammatory Cytokine in Mammary Gland

**Figure 1:**
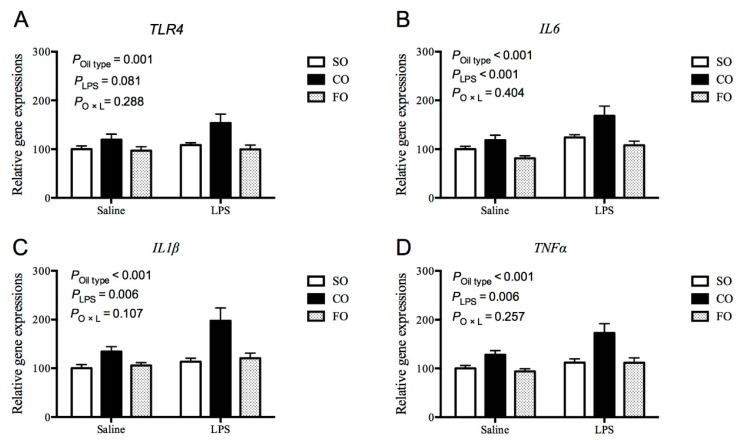
Effect of dietary fat sources during late gestation on mRNA expression of proinflammatory cytokines in mammary gland of lipopolysaccharide-challenged sow. (**A**) *TLR4*, toll-like receptor 4; (**B**) *IL6*, interleukin 6; (**C**) *IL1β*, interleukin 1β; (**D**) *TNFα*, tumor necrosis factor α. Data are represented as means ± standard error (n = 10). SO, group fed the diet supplemented with soybean oil; CO, group fed the diet supplemented with coconut oil; FO, group fed the diet supplemented with fish oil.

**Figure 2:**
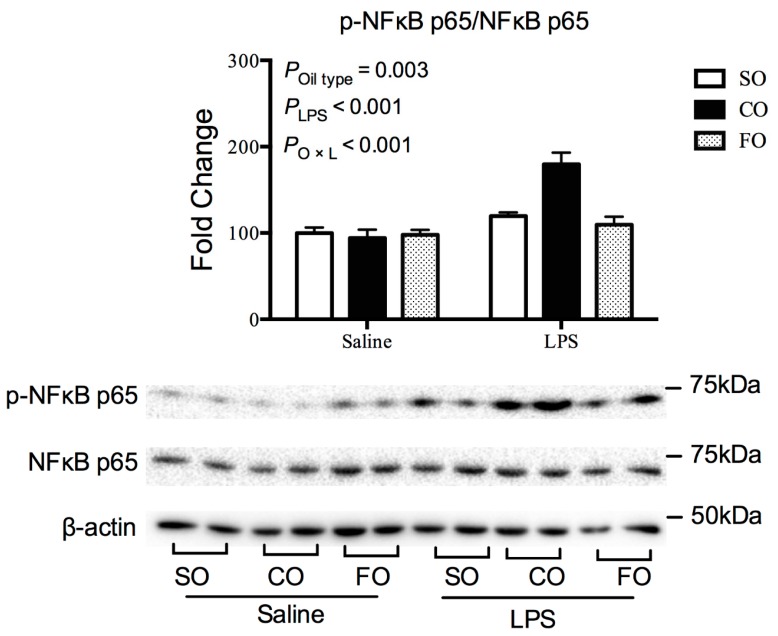
Effect of dietary fat sources during late gestation on nuclear factor-κB inflammatory pathway in mammary gland of lipopolysaccharide-challenged sow. p-NFκB p65, phosphorylated NFκB p65; NFκB p65, nuclear factor κB p65. Data are represented as means ± standard error (n = 10); SO, group fed the diet supplemented with soybean oil; CO, group fed the diet supplemented with coconut oil; FO, group fed the diet supplemented with fish oil.

To explore why LPS-challenged sows fed FO diets exhibited lower colostrum cytokine levels, we further determined the gene expression of inflammatory cytokine in mammary glands. As shown in Figure 1, compared with the SO group, LPS-challenged sows fed CO diet had higher mRNA abundances of toll-like receptor (*TLR4*), *IL6*, *IL1β* and *TNFα* in mammary gland cells (*p* < 0.01), whereas sows fed the FO diet had similar levels of mRNA expression of inflammatory cytokine. Regardless of maternal diet, the mRNA abundance of *IL6*, *IL1β* and *TNFα* in the mammary glands of LPS-challenged sows were increased compared to the saline-treated group (*p* < 0.01). Moreover, LPS challenge increased the phosphorylation level of *p65*, a key component of NF-κB inflammatory pathway (*p* < 0.01, Figure 2). However, the responses of these variables to LPS treatment were lower in sows fed the FO diet compared with the CO group (*p* < 0.01).

## 4. Discussion

The present study used an LPS immune challenge to stimulate a bacterial infection in late-gestating sows to test the hypothesis that bacterial-induced stress can alter the differentiation of mammary epithelial cells and colostrum synthesis, and to investigate the role of maternal diets supplemented with FO or CO on bacterial-induced mammary gland injury. Maternal FO or CO diets did not affect the average weight gain of piglets during the first 24 h period after the onset of parturition compared with the SO diets, which is similar to previous reports showing that maternal FO or CO administration had no significant effect on the growth performance of piglets [3,27,28]. The piglets from LPS-challenged sows had lower average weight gain at postnatal 24 h than those from saline control sows. This is consistent with a previous study in pregnant mice [29]. The yield and composition of colostrum and milk are of greatest importance for piglets’ growth performance [30]. In this study, maternal LPS challenge decreased the colostrum yield and dry matter content. The damaged milking capacity and reduced colostrum dry matter content may contribute to the decreased growth of suckling piglets. As expected, supplementation of FO or CO to gestation diets did not affect colostrum fat content because diets contained less than 5% added fats. In addition, maternal FO diets appeared to alleviate LPS-induced decreased weight gain of piglets and colostrum production, although these effects were not statistically significant, suggesting that feeding FO to sows as a source of n-3 PUFA may play an important role in regulating prenatal maternal stress. Similarly, a recent study demonstrated that maternal n-3 PUFA dietary supplementation attenuated the sow fever response to inflammatory stress challenges [14].

The mammary gland is a highly specialized organ which has tremendous synthetic and secretory capabilities. Mammary bacterial infection contributes to an inflammatory response characterized by injured secretory cell activity, decreased milk yield, and alterations in milk composition [31]. Bacterial LPS endotoxin has been widely used to develop animal models of inflammation [32]. In this study, the ultimate outcome of mammary gland infection is accompanied by the secretion of proinflammatory cytokines such as *TNFα*, *IL1β* and *IL6* in colostrum. In addition, our results indicate that LPS-induced inflammation activated the NF-κB signaling and up-regulated the expression of inflammatory markers in mammary glands. Dietary intervention is an effective method for the treatment of inflammation and related metabolic disorders [33]. NF-κB (p65) is the main pathway in the inflammation cascades, which can be inhibited by n-3 PUFAs such as eicosahexaenoic acid (EPA, C20:5 n-3) and docosahexaenoic acid (DHA, C22:6 n-3) [34,35]. It is reported that dietary FO supplementation could exert a beneficial effect on anti-inflammatory activity [36,37]. Moreover, maternal FO supplementation potentiated an anti-inflammatory response in suckled piglets and accelerated the piglet immune system maturation [38,39]. Also, a recent study demonstrated that maternal supplementation with FO modulated inflammation-related microRNAs and genes in sucking lambs [40]. In the present study, we found that FO inclusion in the sow gestation diet exerted anti-inflammatory effects in mammary glands of sows and thus counteracted the LPS-induced pro-inflammatory response. However, the responses of these variables to LPS treatment were not found in sows fed CO diets.

Sow dietary fat sources affect litter growth performance, and different fatty acids have different effects on sow colostrum or inflammatory response. This experiment selected three oils, namely SO, FO and CO, which differed in fatty acid composition with different carbon chain lengths and number of carbon–carbon double bonds. PUFAs are rich in SO (C18:2 n-6) and FO (C20:5 n-3 and C22:6 n-3), whereas CO contains a large amount of the saturated fatty acids C12:0 and C14:0. Dietary PUFAs have been reported to be beneficial in oxidative stress, lipid metabolism modification and inflammation [41,42,43]. In particular, n-3 PUFA rich in DHA and EPA is a major contributor to inhibiting inflammation [40]. On the contrary, saturated fatty acids trigger a TLR4-mediated inflammatory response [44,45]. A recent study demonstrated that virgin CO is associated with high-fat diet-induced metabolic alterations and adipose inflammation in rats [46]. Similarly, the present study showed the negative effect of CO supplementation to sows on LPS-induced mammary inflammation. Compared with CO diets, dietary supplementation with FO or SO protected sows from LPS challenge-induced mammary inflammatory responses, as indicated by decreased pro-inflammatory cytokines expression and *p65* phosphorylation level. It is noteworthy to mention that the maternal dietary inclusion of FO in the current study may increase DHA and EPA proportions in sow colostrum and serum. Therefore, we conclude that the inflammatory response in the mammary glands of sows challenged with LPS was affected by the different carbon chain lengths of the fatty acids. However, as the fatty acid compositions of sow colostrum and piglet serum were not explored in the current study, further investigation is required to fully understand how dietary FO inclusion improved mammary gland inflammation and piglet growth.

## 5. Conclusions

Maternal LPS challenge induced a mammary inflammatory response and damaged colostrum production and piglet growth. The inclusion of FO in sow gestation diets exerted anti-inflammatory effects in the mammary glands of sows and counteracted the LPS-induced pro-inflammatory response when compared to CO. These results may provide a strategy to attenuate maternal stress and inflammation and maintain immune balance during gestation. Future analysis including fatty acid profiles of sow colostrum and piglet serum and tissue-specific gene expression may help to explain how maternal LPS challenge and FO inclusion influence the metabolic health of sows and their progeny.

## Figures and Tables

**Table 1:** Ingredients and composition of the experimental diets (as-fed basis).

Ingredients (g/kg of Diet)	Soybean Oil	Coconut Oil	Fish Oil
Corn	645.0	645.0	645.0
Soybean meal (CP44%)	130.0	130.0	130.0
Wheat bran	150.0	150.0	150.0
Soybean oil	30.0	-	-
Fish oil	-	-	30.0
Coconut oil	-	30.0	-
Fish meal (CP65%)	20.0	20.0	20.0
Lysine-HCl (98.5%)	0.3	0.3	0.3
DL-Methionine (99%)	0.3	0.3	0.3
L-Threonine (99%)	0.4	0.4	0.4
Limestone	6.5	6.5	6.5
Monocalcium phosphate	10.0	10.0	10.0
NaCl	4.0	4.0	4.0
Choline chloride (50%)	1.0	1.0	1.0
Vitamin mix ^1^	0.5	0.5	0.5
Mineral mix ^2^	2.0	2.0	2.0
Total	1000.0	1000.0	1000.0
Calculated composition			
Digestible energy, Mcal/kg	3.30	3.26	3.30
Crude protein, %	14.25	14.25	14.25
Ca, %	0.72	0.72	0.72
Total P, %	0.68	0.68	0.68
Available P, %	0.41	0.41	0.41
Total lysine, %	0.62	0.62	0.62
Total methionine, %	0.23	0.23	0.23
Total threonine, %	0.48	0.48	0.48
Total tryptophan, %	0.13	0.13	0.13

^1^ The vitamin mix provided (per kg of complete diet): vitamin A, 4000 IU; vitamin D_3_, 800 IU; vitamin E, 44 IU; vitamin K_3_, 0.5 mg; Thiamine, 1 mg; Riboflavin, 3.75 mg; vitamin B6, 1 mg; vitamin B_12_, 15 μg; Niacin, 10 mg; Biotin, 0.2 mg; Pantothenic acid, 12 mg; Folic acid, 1.3 mg. ^2^ The mineral premix provided the following per kg of complete diet: Cu 10 mg as CuSO_4_·5H_2_O; Fe 80 mg as FeSO_4_; I 0.6 mg as KI; Zn 100 mg as ZnSO_4_; Mn 25 mg as MnSO_4_; Se 0.15 mg as Na_2_SeO_3_.

**Table 2:** Fatty acid composition of soybean oil, coconut oil and fish oil (g/100g fatty acids).

	Soybean Oil	Coconut Oil	Fish Oil
Ether extract, %	99.64	99.86	99.56
Fatty acids, % of ether extract			
C12:0	1.13	42.89	0.23
C14:0	0.26	23.44	6.58
C16:0	12.36	8.36	15.33
C16:1 (n-7)	0.31	0.12	7.06
C18:0	4.63	2.56	3.85
C18:1 (n-9)	20.16	8.74	16.06
C18:2 (n-6)	53.63	0.03	4.55
C18:3 (n-3)	7.56	1.22	2.26
C20:0	0.53	0.34	0.82
C20:1 (n-9)	not detected	0.54	2.04
C20:5 (n-3)	0.23	not detected	16.89
C22:1	not detected	not detected	3.31
C22:5 (n-3)	not detected	not detected	1.96
C22:6 (n-3)	0.38	not detected	13.99

**Table 3:** Nucleotide sequences of primers used to measure targeted genes.

Gene Symbols	Nucleotide Sequence of Primers (5′-3′)	Accession No.
*ACTB*	F: TCTGGCACCACACCTTCT R: TGATCTGGGTCATCTTCTCAC	XM_003124280.3
*TOP2B*	F: AACTGGATGATGCTAATGATGCT R: TGGAAAAACTCCGTATCTGTCTC	NM_001258386.1
*TBP*	F: GATGGACGTTCGGTTTAGG R: AGCAGCACAGTACGAGCAA	DQ178129
*TLR4*	F: TCAGTTCTCACCTTCCTCCTG R: GTTCATTCCTCACCCAGTCTTC	GQ503242.1
*IL6*	F: GACAAAGCCACCACCCCTAA R: CTCGTTCTGTGACTGCAGCTTATC	M80258.1
*IL1β*	F: TCTGCCTGTACCCCAACTG R: CCAGGAAGACGGGCTTTTG	NM214055.1
*TNFα*	F: CGTGAAGCTGAAAGACAACCAG R: GATGGTGTGAGTGAGGAAAACG	EU682384.1

*ACTB*, β-actin; *TOP2B*, DNA topoisomerase Ⅱ beta; *TBP*, TATA-binding protein; *TLR4*: toll like receptor 4; *IL*6: interleukin 6; *IL1β*: interleukin 1β; *TNF*α: tumor necrosis factor α.

**Table 4:** Effect of dietary fat sources during late gestation on reproductive performance of lipopolysaccharide (LPS) -challenged sows (n = 10 per group).

Items	Soybean Oil		Coconut Oil		Fish Oil			*p*-Value		
	Saline	LPS	Saline	LPS	Saline	LPS	SEM	Oil Type	LPS	O × L
Sow body weighgt at day 90, kg	261.3	262.1	263.0	262.9	260.0	261.1	4.6	0.891	0.885	0.992
Sow backfat at day 90, mm	14.6	14.9	15.0	15.0	15.0	14.9	0.4	0.862	0.823	0.920
Duration of farrowing, h	4.3	3.4	4.1	3.9	3.5	4.2	0.9	0.980	0.751	0.478
Onset of transient milk production, h	30.2	31.3	31.2	30.4	33.9	32.8	1.3	0.125	0.821	0.698
Litter size, n										
Total born	12.3	12.0	12.6	13.0	12.5	12.6	1.2	0.857	0.972	0.966
Born alive	11.2	11.3	11.1	11.4	11.3	11.0	1.3	0.981	0.950	0.987
Mortality from day 1 to 5, %	4.3	3.7	3.9	5.2	5.6	4.5	0.7	0.431	0.991	0.198
Piglets										
Birth weight, kg	1.45	1.52	1.47	1.50	1.39	1.42	0.1	0.816	0.706	0.994
Weight gain (0 to 24 h), g	132	109	132	94	129	114	10	0.497	<0.001	0.357

**Table 5:** Effect of dietary fat sources and lipopolysaccharide (LPS) stimulation during late gestation on colostrum yield of sows and colostrum intake by piglets (n = 10 per group).

Items	Soybean Oil		Coconut Oil		Fish Oil			*p*-Value		
	Saline	LPS	Saline	LPS	Saline	LPS	SEM	Oil Type	LPS	O × L
0 to 24 h Colostrum yield (kg sow^-1^)	4.4	3.6	5.1	3.8	5.0	4.7	0.3	0.084	0.014	0.437
0 to 24 h Colostrum intake (g piglet^-1^)	392	317	458	323	441	424	36	0.059	0.005	0.189

**Table 6:** Effect of dietary fat sources during late gestation on chemical composition of colostrum from lipopolysaccharide (LPS)-challenged sows (n = 10 per group).

Items	Soybean Oil		Coconut Oil		Fish Oil			*p*-Value		
	Saline	LPS	Saline	LPS	Saline	LPS	SEM	Oil Type	LPS	O × L
Protein (%)	10.8	9.1	10.9	9.3	10.4	10.2	0.8	0.964	0.270	0.808
Fat (%)	5.3	4.9	5.4	5.1	5.6	5.4	0.6	0.733	0.492	0.996
Lactose (%)	4.4	4.3	4.7	4.6	4.5	4.3	0.2	0.218	0.372	0.951
Dry matter (%)	22.4	20.2	22.3	19.1	21.9	20.8	1.0	0.759	0.005	0.502

**Table 7:** Effect of dietary fat sources during late gestation on cytokines concentrations in colostrum from lipopolysaccharide (LPS)-challenged sows (n = 10 per group) ^1^.

Items	Soybean Oil		Coconut Oil		Fish Oil			*p*-Value		
	Saline	LPS	Saline	LPS	Saline	LPS	SEM	Oil Type	LPS	O × L
*IL6* (mg/L)	1.23	2.23	2.12	4.05	0.98	1.17	0.4	<0.001	<0.001	<0.001
*IL1β* (ng/L)	23.13	43.67	29.91	75.78	20.45	33.11	5.4	<0.001	<0.001	<0.001
*TNFα* (ng/L)	121.3	149.4	109.4	229.4	119.4	129.3	24.5	0.009	<0.001	<0.001

^1^*IL6*: interleukin 6; *IL1β*: interleukin 1β; *TNFα*: tumor necrosis factor α.
